# Simple steps to avoid plagiarism and improve scientific writing

**DOI:** 10.3402/ljm.v8i0.21825

**Published:** 2013-07-25

**Authors:** Syed Wali Peeran, Aisha Mojtaba Ahmed, Marei Hamed Mugrabi, Syed Ali Peeran

**Affiliations:** 1 Faculty of Dentistry, Department of Periodontology and Oral Implantology, Sebha University, Sebha, Libya; 2 Faculty of Medicine, Sebha University, Sebha, Libya; 3 Faculty of Dentistry, Department of Periodontics, Arab Medical University, Benghazi, Libya; 4 Department of Oral and Maxillofacial Prosthodontics, Faculty of Dentistry, Jazan University, Jazan, KSA

Dear Sir,

Plagiarism is defined by the Oxford dictionary as ‘the practice of taking someone else's work or ideas and passing them off as one's own’ (1). Plagiarism can be defined simply as literary theft. Historically, it used to take place when one tried to steal other's work to gain recognition. In the recent times, plagiarism includes literary theft or misappropriation of intellectual property and the substantial unattributed textual copying of another's work (2). Plagiarism encompasses either plagiarizing from others or republishing substantial parts of one's own work without citing the same, as separate new works.

Publications are the endpoints and fruits of research projects that are meticulously planned and executed. Unfortunately in the developing world, on the one hand, we lack the means of proper research training for new students, academicians, clinicians, and researchers, due to financial constraints (3). On the other hand, regulating authorities do not have alternative mechanisms to evaluate the professional standing of an individual except an individual's publications. Hence, an undue pressure to publish exists. Also, the lack of a clear idea and understanding of what plagiarism actually is and the consequences that follow upon attempting it often lead one to plagiarize.

A relatively recent term, salami publication, has also become common. This is when one divides their research work into small, inappropriate parts and gets them published. It is worth noting that a good and decent work in a reputed peer-reviewed journal is more worthy than small works, which run the risk of being withdrawn by the editors/journals if found to have breached the desirable publication ethics.

Following these simple steps will be of much benefit:

1. If you lack expertise or if you do not know how to plan and execute your research work and draft the manuscript, then join a group of experienced researchers who are working on a few projects. Working with an experienced team will provide you with a vast amount of knowledge and expertise.
2. Before submitting your manuscript for publication, ask your peers who have already published their works to check it. Simple suggestions may be both timely and of great help.
3. Cite correctly and adequately, as necessary. One must know that citations do not reduce the value of one's article.
4. Pass your prepared manuscript through plagiarism check websites before you submit. This will give you an instant idea of where your manuscript stands and can often save someone from embarrassment of being caught plagiarizing. A few examples include:
  - http://www.ithenticate.com/
  - http://www.duplichecker.com/
  - http://smallseotools.com/plagiarism-checker/
  - http://www.plagium.com/
  - www.articlechecker.com
5. Submit your manuscript for scientific language editing as and when required. The following includes some examples of scientific language editorial sites:
  - http://webshop.elsevier.com/languageediting/
  - https://sites.google.com/site/makeditors/
  - http://www.medilinkers.com/consultancy.htm
6. You can also improve your illustrations, create 3-/4-dimensional illustrations, line diagrams, and flow charts using various web-based illustration services. This will improve the chances of your manuscript being accepted. Some examples include:
  - http://webshop.elsevier.com/illustrationservices/
  - http://www.stromastudios.com/services.html#illustration

Remember, not having a publication to your credit or having fewer publications than others does not mean that you are less professional than your colleagues. It just means that you have not given serious thought to do genuine research. And there are no shortcuts to success.

This letter is a sincere attempt to help naïve researchers and We hope will be of a nudge in the right direction toward developing their skills, helping them understand the pitfalls in their paths and the simple means to mend them off.

*Syed Wali Peeran* Faculty of Dentistry Department of Periodontology and Oral Implantology Sebha University Sebha, Libya *Aisha Mojtaba Ahmed* Faculty of Medicine Sebha University Sebha, Libya
*Marei Hamed Mugrabi* Faculty of Dentistry Department of Periodontics Arab Medical University Benghazi, Libya *Syed Ali Peeran* Department of Oral and Maxillofacial Prosthodontics Faculty of Dentistry, Jazan University Jazan, KSA
